# Plasma kallikrein structure reveals apple domain disc rotated conformation compared to factor XI

**DOI:** 10.1111/jth.14418

**Published:** 2019-03-19

**Authors:** Chan Li, Kayleigh M. Voos, Monika Pathak, Gareth Hall, Keith R. McCrae, Ingrid Dreveny, Renhao Li, Jonas Emsley

**Affiliations:** ^1^ Centre for Biomolecular Sciences School of Pharmacy University of Nottingham Nottingham UK; ^2^ Aflac Cancer and Blood Disorders Center Department of Pediatrics Emory University School of Medicine Atlanta GA USA; ^3^ Departments of Hematology and Oncology and Cellular and Molecular Medicine Cleveland Clinic Cleveland OH USA

**Keywords:** factor IX, factor XI, factor XII, kininogens, plasma kallikrein

## Abstract

**Essentials**

- Zymogen PK is activated to PKa and cleaves substrates kininogen and FXII contributing to bradykinin generation.
- Monomeric PKa and dimeric homologue FXI utilize the N‐terminal apple domains to recruit substrates.
- A high‐resolution 1.3 Å structure of full‐length PKa reveals an active conformation of the protease and apple domains.
- The PKa protease and four‐apple domain disc organization is 180° rotated compared to FXI.

**Summary:**

**Background**

Plasma prekallikrein (PK) and factor XI (FXI) are apple domain‐containing serine proteases that when activated to PKa and FXIa cleave substrates kininogen, factor XII, and factor IX, respectively, directing plasma coagulation, bradykinin release, inflammation, and thrombosis pathways.

**Objective**

To investigate the three‐dimensional structure of full‐length PKa and perform a comparison with FXI.

**Methods**

A series of recombinant full‐length PKa and FXI constructs and variants were developed and the crystal structures determined.

**Results and conclusions**

A 1.3 Å structure of full‐length PKa reveals the protease domain positioned above a disc‐shaped assemblage of four apple domains in an active conformation. A comparison with the homologous FXI structure reveals the intramolecular disulfide and structural differences in the apple 4 domain that prevents dimer formation in PK as opposed to FXI. Two latchlike loops (LL1 and LL2) extend from the PKa protease domain to form interactions with the apple 1 and apple 3 domains, respectively. A major unexpected difference in the PKa structure compared to FXI is the 180° disc rotation of the apple domains relative to the protease domain. This results in a switched configuration of the latch loops such that LL2 interacts and buries portions of the apple 3 domain in the FXI zymogen whereas in PKa LL2 interacts with the apple 1 domain. Hydrogen‐deuterium exchange mass spectrometry on plasma purified human PK and PKa determined that regions of the apple 3 domain have increased surface exposure in PKa compared to the zymogen PK, suggesting conformational change upon activation.

## Introduction

The regulation of extracellular proteolysis is critical to blood hemostasis and innate immunity and proteases plasma prekallikrein (PK), and coagulation factors XII (FXII) and XI (FXI) act as master regulators of several cascades including coagulation, fibrinolysis, complement activation, and inflammation 1, 2, 3, 4. PK is an unusual plasma protease as it does not recruit its principal substrate high‐molecular‐weight kininogen (HK) upon activation but rather it circulates as a zymogen in a tightly bound but inactive PK‐HK complex 5, raising the question as to how this complex is effectively regulated. The PK zymogen can be activated by a single cleavage from two‐chain factor XIIa (FXIIa) and can also be activated with reduced efficiency by single‐chain FXII 6.

The PK‐HK complex can undergo autoactivation in the presence of zinc ions and negatively charged polymers 7, 8 including polyphosphates and nucleic acids 9. Prekallikrein has been shown to bind to HK via the apple 2 domain 10. The active form plasma kallikrein (PKa) performs a double cleavage of HK to liberate the vasoactive peptide bradykinin, which binds the G‐protein coupled receptor (GPCR) B2R on endothelial cells leading to activation of signaling events. Deficient regulation of the system is exemplified by the disorder hereditary angioedema where reduction of the natural inhibitor serpin C1 or a hyperactivated FXII results in excessive bradykinin generation and recurrent attacks of severe tissue swelling 11. PKa is currently being targeted as a novel means to treat diverse thrombotic 12, 13, 14 and inflammatory disorders such as diabetic macular edema 15, 16, desquamating skin diseases, and neurodegenerative disorders 12, 17.

**Figure 1:**
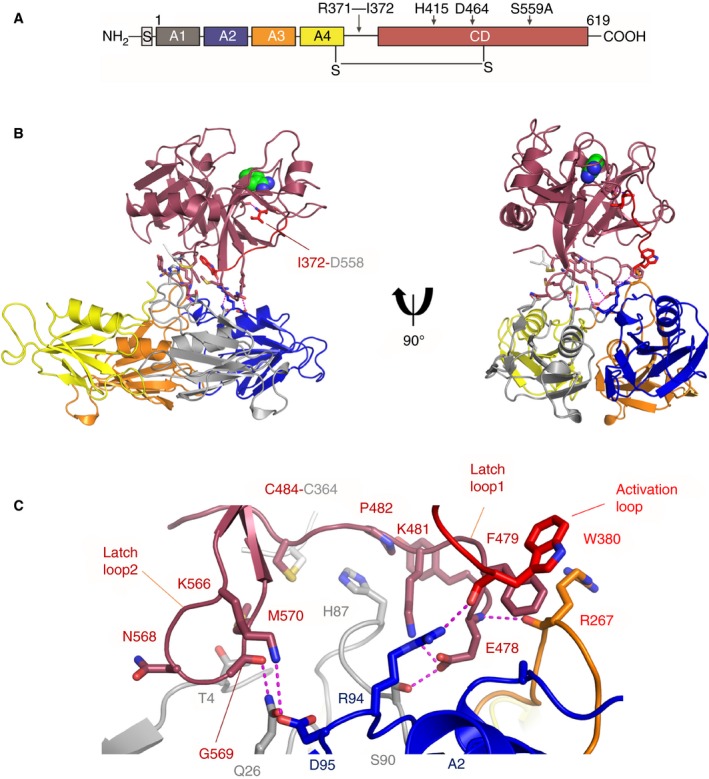
Structure of PKa. (A) Domain organization of plasma prekallikrein. The Arg371‐Ile372 cleavage site in the activation loop is indicated. (B) A cartoon diagram of the PKa structure shows two views related by a 90°‐rotation with the protease domain in dark red and the four apple domains colored as A1 (gray), A2 (blue), A3 (orange), and A4 (yellow). Benzamidine is shown as spheres (green) bound in the S1 pocket. The activation loop is red with Ile372 shown as sticks salt bridging to Asp558. Interfacial residues are shown as sticks with electrostatic and hydrogen bonds as dotted purple lines. (C) Close‐up view of the interface between the PKa protease domain and apple domain disc.

PK and FXI are homologous and share 58% amino acid sequence identity with the unique feature of four tandem repeats of 90 amino acid apple domains at the N‐terminus (Fig. 1A), which harbor exosites for substrates, cofactors, and receptors 18. We have previously described the crystal structure for the inactive FXI zymogen 19 and a model for the PK zymogen 20. Factor XI is a disulfide‐linked dimer whereas PK is a monomer 20, 21. The important active form of FXI (FXIa) has been described as half‐activated whereby one side of the dimer remains as a zymogen with the activation loop intact whereas the other side is cleaved 22. In FXIa the apple 3 domain has a binding site for the Gla domain from substrate Factor IX (FIX) and it has been shown that this is cryptic in the zymogen FXI 23, 24, 25. Factor XIIa recognizes and cleaves both PK and FXI but it is unknown which of the apple domains is critical for these interactions or the molecular basis of the reciprocal cleavage of the FXII zymogen by PKa. Here we report the active conformation of the full‐length PKa defined at high resolution and identify large conformational and organizational rearrangements compared to the structure of the FXI zymogen.

## Methods

### Protein purification, crystallization, and structure determination

A gene encoding human PK (gene *KLKB1*) was cloned into the pMT‐puro vector using BglII and MluI restriction sites with a six‐histidine tag at the N‐terminus after the immunoglobulin binding chaperone protein secretion signal for expression using the *Drosophila* expression system (Invitrogen, Carlsbad, CA, USA). A series of constructs and mutants were evaluated to improve recombinant PK for crystallization purposes. The final recombinant PK variant utilized site‐directed mutagenesis (SDM) (QuikChange, Agilent Technologies, santa clara, CA, USA) to mutate two high‐entropy cluster residues E323A, K325A, K507A, and K509A, and three glycosylation sites N377Q, N434Q, and N475Q on the catalytic domain, together with the active site mutant S559A to prevent autoproteolysis and optimize PK for crystallization experiments (residue numbering corresponds to the mature PK protein with signal peptide removed).

*Drosophila* S2 cells were grown in Dulbecco's modified eagle's medium (Gibco) supplemented with 10% fetal calf serum at 28 °C and transfection was performed using calcium phosphate. Cells were grown for an additional 48 h before selection with puromycin to establish stable cell lines. Proteins were expressed in serum‐free Express Five insect culture medium (Gibco) and the cells were harvested 6 days after induction with 0.5 mm CuSO_4_. Protein purification was performed using a Capto S column followed by a Ni‐sepharose affinity column and a Superdex 200 gel filtration column (GE healthcare, Boston, MA, USA).

The activated form PKa was prepared by incubating pure recombinant PK (1 mg mL^−1^) with *β*‐FXIIa at a ratio of 40 : 1 in 20 mm Tris‐Cl, 150 mm NaCl, pH 8 overnight at 20 °C. The reaction was stopped by adding benzamidine at a final concentration of 20 mm. PKa was further purified by gel filtration (Superdex 200 column) and then concentrated to 8.2 mg mL^−1^. Crystallization was performed at 20 °C and 10 °C using sparse matrix screens (Hampton Research, Molecular Dimensions, Qiagen, Venlo, Netherlands) in sitting drop plates. Crystals were observed for PKa in the Morpheus screen with a reservoir containing 0.1 m carboxylic acids, 0.1 m Hepes/MOPS pH 7.5, 12.5% MPD, 12.5% PEG1000, 12.5% PEG3350 and a drop containing 0.1 m carboxylic acids, 0.1 m Tris/BICINE pH 8.5, 20% PEG 500 MME, 10% PEG 20 000. Recombinant FXI was a gift from Novo Nordisk and crystallized as described previously 26. Single crystals were transferred to the reservoir solution containing 20% glycerol, and flash cooled in liquid nitrogen. Diffraction data were collected at Diamond beam line I04 to 1.3 Å for PKa, and the European Synchrotron Radiation Facility (ESRF) beamline ID4 for the 2.6 Å FXI zymogen structure. Data were processed and reduced using XDS and the CCP4 suite 27. The structures were determined by molecular replacement (PHASER 28) using coordinates from the FXI heavy chain (PDB code 2F83 19) and PK catalytic domain (PDB code 2ANW 29) as search models. Models were built with COOT 30 and refined with REFMAC. Data collection and refinement statistics are summarized in Table 1. Figures were generated using PyMOL. A homology model of the PK zymogen based on the FXI structure described in Table 1 was generated using SWISSMODEL 31, which improved the previous model [20.

**Table 1:** Data collection and structure refinement statistics

	PKa	FXI
Data collection		
Space group	P2_1_2_1_2	P4_3_2_1_2
Cell dimensions		
*a*,* b*,* c* (Å)	90.6, 129.6, 55.8	80.8, 80.8, 251.1
*α*,* β*,* γ* (°)	90, 90, 90	90, 90, 90
Wavelength (Å)	0.9762	0.9334
Resolution (Å)	1.3	2.6
*R*_merge_ *	0.05 (0.682)	0.09(0.602)
I/σI	18.1 (2.5)	20.7 (2.8)
Completeness (%)	99.8 (98.0)	99.3 (96.9)
Multiplicity	6.6 (6.5)	8.8 (6.7)
CC ½	0.999 (0.76)	0.995 (0.72)
Unique reflections	141 636	26 378
Structure refinement		
*R*_work_	0.164 (0.253)	0.208 (0.315)
*R*_free_	0.196 (0.257)	0.281 (0.416)
Overall B factor (A^2^)	20.0	72.4
Stereochemical r.m.s.d.		
Bond length (Å)	0.033	0.013
Bond Angle (°)	2.82	1.77
Ramachandran statistics†		
Favoured (%)	98.1	94.1
Allowed (%)	1.9	5.0
Outliers (%)	0	0.9

*The numbers in parentheses are for the highest‐resolution shell. †Ramachandran statistics are calculated using Molprobity. FXI, factor XI; PKa, plasma kallikrein; r.m.s.d., root mean square deviation.

### Hydrogen deuterium exchange mass spectrometry

Hydrogen deuterium exchange mass spectrometry (HDX‐MS) was performed largely as described 32 on a Waters HDX system with nanoAcquity UPLC (Waters, Milford, MA, USA) and Micromass Q‐ToF Premier mass spectrometer. Samples were measured in tandem using the same buffers to minimize the difference in back exchange. Samples at 1.5 mg mL^−1^ of human plasma purified PK and PKa supplied by Enzyme Research Laboratories (termed HPK and HPKa) were diluted 1 : 7 (v : v) into labeling buffer (10 mm phosphate, 99.9% D_2_O, pD 7.0) for 10 to 10 000 s at 20 °C by automated LEAP robot pipetting. The 0‐s time point was represented by the dilution into the H_2_O‐based labeling buffer. The HDX was quenched 1 : 1 (v : v) with precooled buffer containing 100 mm phosphate, 0.5 m TCEP, 0.8% formic acid, 2% acetonitrile, pH 2.5 for 180 s at 1 °C and digested on a Waters Enzymate BEH Pepsin Column (2.1 × 30 mm) at 20 μL min^−1^. Fragments were separated on a Waters Nano ACQUITY UPLC BEH C18 column (1.7 μm, 1.0 × 100 mm) at 40 μL min^−1^ with a gradient of 40% to 90% acetonitrile. Mass spectrometry was performed using electrospray ionization in positive ion mode. Peptides with no exchange were sequenced via Protein Lynx Global Software V3.0.2, followed by amide deuterium uptake analysis done manually via Dynamx 3.0 software. Fragments with >0.2‐Da mass deviations were removed.

## Results

### Structure of full‐length plasma kallikrein

To determine a full‐length PKa structure recombinant optimized PK active site mutant S559A was activated by proteolytic cleavage of the activation loop Arg371‐Ile372 using FXIIa. PKa crystallized in the presence of inhibitor benzamidine and the structure was determined to 1.3 Å resolution (Table 1). Figure 1A shows the domain architecture and Fig. 1B the overall structure with the protease domain positioned on top of the disc‐shaped section of the four‐apple domains.

The PKa protease structure consists of two *β*‐barrels and the benzamidine inhibitor is observed bound in the active site S1 pocket with the Ile372 residue from the activation loop forming the characteristic electrostatic interaction with Asp558 (Fig. 1B). The full‐length structure defines the interactions formed between the PKa protease domain and the apple domains whereby two extended loops “latch” on to the apple domains with specific interactions shown in Fig. 1C. Residues 476 to 484 form a latch loop1 and residues 566 to 570 comprise latch loop2. The two latch loops project from the protease N‐terminal and C‐terminal *β*‐barrels, respectively, forming contacts with residues from loops on the upper face of apple domains 1 to 3 (Fig. 1C). Latch loop1 contains a central *β*‐hairpin that inserts the salt bridged residues Glu478‐Lys481 into a pocket at the center of the apple domain disc where Glu478 forms hydrogen bonds with Arg267 and Ser90 (Fig. 1C). Latch loop1 residues Pro482, Tyr476 and the Cys364‐Cys484 disulfide form a pocket that encircles His87 from apple domain 1. Latch loop2 adopts an extended *β*‐hairpin and on its tip residue Lys566 forms a salt bridge to Asp95 from the apple 2 domain and Gly569 in latch loop2 forms a hydrogen bond to Gln26 from the apple 1 domain topside α‐helix. Additional contacts between the base of the activation loop and Arg94, which forms a hydrogen bond to Trp380, are observed. These three contact points encircle a central cavity between the protease and apple domain disc that is filled with ordered water molecules.

### Comparison of the PKa apple domains with FXI

**Figure 2:**
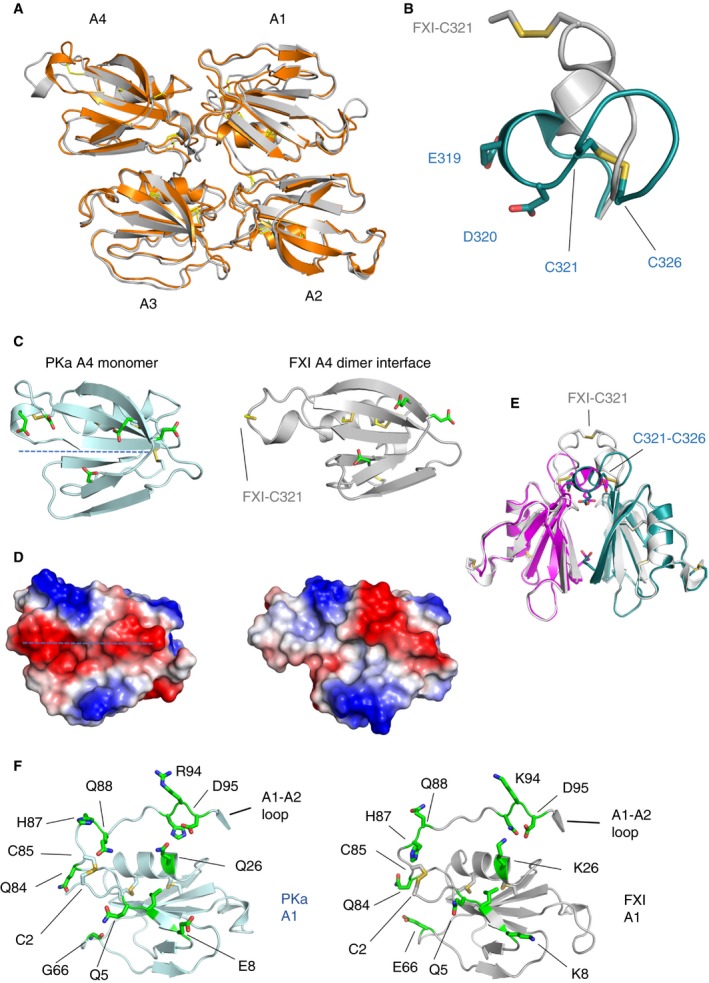
Comparison of the PKa and FXI apple (A1–4) domains. (A) A cartoon diagram of the PKa apple 1 to 4 domain structure is shown colored orange superposed onto the FXI apple domain structure in gray. (B) A comparison of PKa (cyan) and FXI (gray) residues 321 to 326 superposed showing the intramolecular disulfide unique to PKa. (C) A cartoon diagram highlighting the altered conformation of PKa apple 4 domain (left), which shows the internal Cys321‐Cys326 disulfide together with residues Glu319, Asp320. These structural features are not present in FXI (right). (D) Charged surface representation of the PKa (left) and the FXI (right) apple 4 domain showing the differences in the region of the FXI dimer interface and the introduction of a continuous region of negative charge in PKa (dotted line). (E) Cartoon diagram showing the FXI apple 4 domain dimer (gray) superposed onto the PKa apple 4 domain (magenta and cyan) illustrating steric disruption of the interface by Cys321‐Cys326. (F) The PKa apple 1 domain (light blue on the left) is compared with the FXI apple 1 domain (gray on the right) with surface exposed residues shown as sticks in green and disulfides in yellow. FXI residue Glu66 has been described as forming part of the thrombin binding site and this is not conserved in PK, being replaced by Gly66 consistent with the lack of interaction between thrombin and PK. FXI, factor XI; PKa, plasma kallikrein.

To enhance the quality of the comparison between the two proteins and generate a more accurate model of the PK zymogen we collected improved 2.6 Å resolution crystallographic data from crystals of the FXI zymogen (Table 1). The higher‐resolution structure revealed a number of additional features not observed in the previously reported 3 Å resolution FXI structure 19 including definition of the activation loop residues and loops surrounding the active site. The organization and interfaces between individual apple domains are closely preserved when PKa is compared to FXI 19 with four α‐helices presented on the top surface (as viewed in Fig. 1B) and four *β*‐sheets on the bottom of the disc (Fig. 2A).

An alternate conformation occurs in the apple 4 domain where an internal Cys321‐Cys326 disulfide distinguishes PK as a monomer precluding formation of the extensive disulfide‐linked dimer interface observed in FXI 19. Figure 2B shows a comparison of PK (cyan) and FXI (gray) residues 321 to 326 superposed highlighting the intramolecular disulfide unique to PK. Figure 2C illustrates a comparison with FXI and the altered conformation of PK apple 4 domain with the internal Cys321‐Cys326 disulfide together with residues Glu319, Asp320, both of which are not present in the FXI structure. Major changes in the charged surface of the PK apple 4 domain compared to the FXI apple 4 domain in the region of the FXI dimer interface are highlighted as a dotted line in Fig. 2D. A superposition of the FXI apple 4 domain dimer (gray) onto the PK apple 4 domain (magenta and cyan) illustrates steric disruption of the dimeric interface by an altered conformation, altered charge, and a Cys321‐Cys326 disulfide bridge in the PKa structure (Fig. 2E).

**Figure 3:**
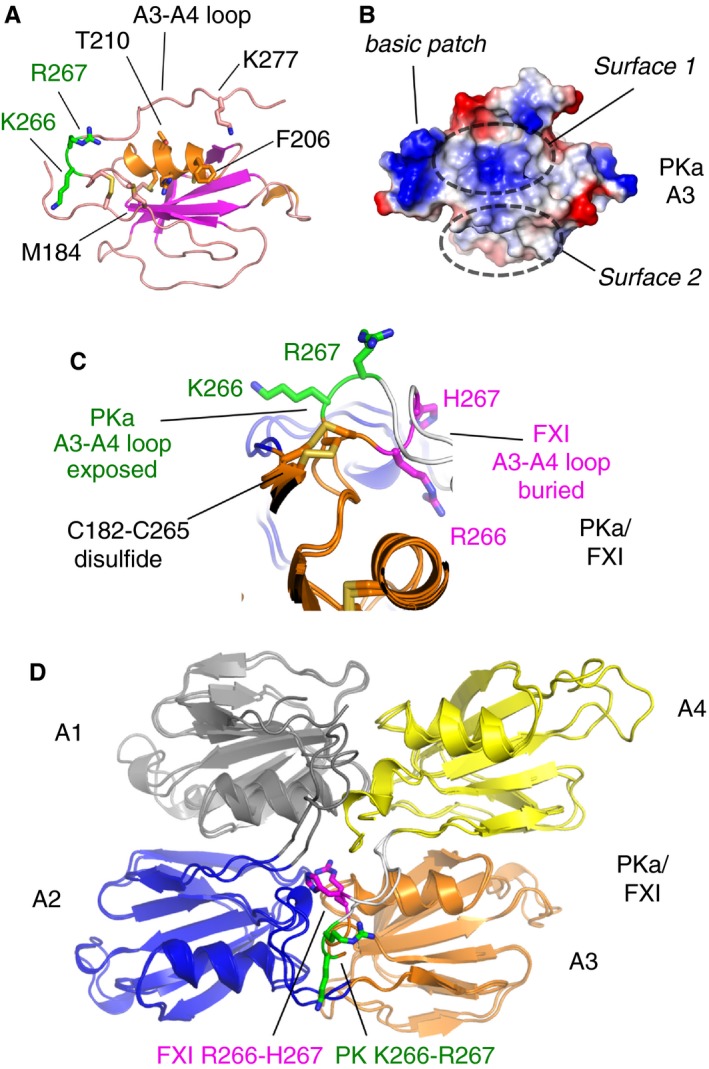
Comparison of the PKa and FXI apple 3 domains. (A) A cartoon diagram of the PKa apple 3 domain structure is shown with key residues highlighted as sticks. (B) Charged representation of the apple 3 domain upper surfaces. A pocket formed by the loop connecting to the apple 4 domain is indicated by a dashed elliptical line labeled as surface 1. A second flat surface 2 is defined by residues from the N‐terminal region of the apple 3 domain. (C) Cartoon diagram showing a superposition of the loop structure connecting the apple 3 and 4 domains from PKa with the equivalent FXI structure. Residues Lys266 and Arg267 (green) from PKa are raised and become surface exposed compared to the equivalent residues His267 and Arg266 in FXI (purple), which are buried. Disulfides are in yellow. (D) Cartoon diagram showing the PKa and FXI apple 1 to 4 domains superposed and colored as in Fig. 1. Key residues showing a major conformational difference in the apple 3 to 4 domain connecting loop are shown as sticks. FXI, factor XI; PKa, plasma kallikrein.

The apple 1 domains of PKa and FXI are shown on the left and right of Fig. 2F, respectively, with the overall conformation preserved. A thrombin binding site has been identified in the apple 1 domain of FXI localized to Glu66, Lys83, and Gln84 33, 34, 35. Factor XI Glu66 is replaced by Gly66 in PKa consistent with the observations that thrombin does not interact with or activate PK. Compared to the apple 1 domain more significant differences occur in the region of the apple 3 domain where a loop connecting the apple 3 and apple 4 domains adopts alternate conformations. In FXI this connecting loop is buried, whereas it becomes exposed onto the surface generating a pocket in PKa (Fig. 3A–C). The largest difference is observed for residues 266 and 267, which are buried in the center of the apple domain disc in FXI (purple in Fig. 3D) but are presented on the surface in PKa where Lys266 and Arg267 combine to form a positively charged patch (Fig. 3B). In the comparison of PKa and FXI the apple 3 domain Cys182‐Cys265 disulfide switches its side chain rotamers significantly to accommodate this conformation change whereas in the apple 1 domain the equivalent Cys2‐Cys85 disulfide remains in one conformation (Fig. 2F).

Figure 3D highlights the apple 1 domain is equivalent to the apple 3 domain in terms of the way the domains are arranged in the disc as the long α‐helix is partially buried between the interdomain loops and is tucked closer to the center of the apple domain disc whereas the equivalent α‐helix in the apple 2 and apple 4 domains is located radially on the surface. This disposition means both the apple 1 and apple 3 domains present two equivalent solvent exposed surfaces labeled surface 1 and 2 in Fig. 3B. In FXI residues from surface 1 have been identified as the apple 3 domain binding site for the substrate FIX Gla domain 25, 36, 37 and surface 2 is the location of the FXI anion binding site whereas the function of these surfaces is unknown in PK 23.

### Differences in the overall organization of PKa compared to FXI

**Figure 4:**
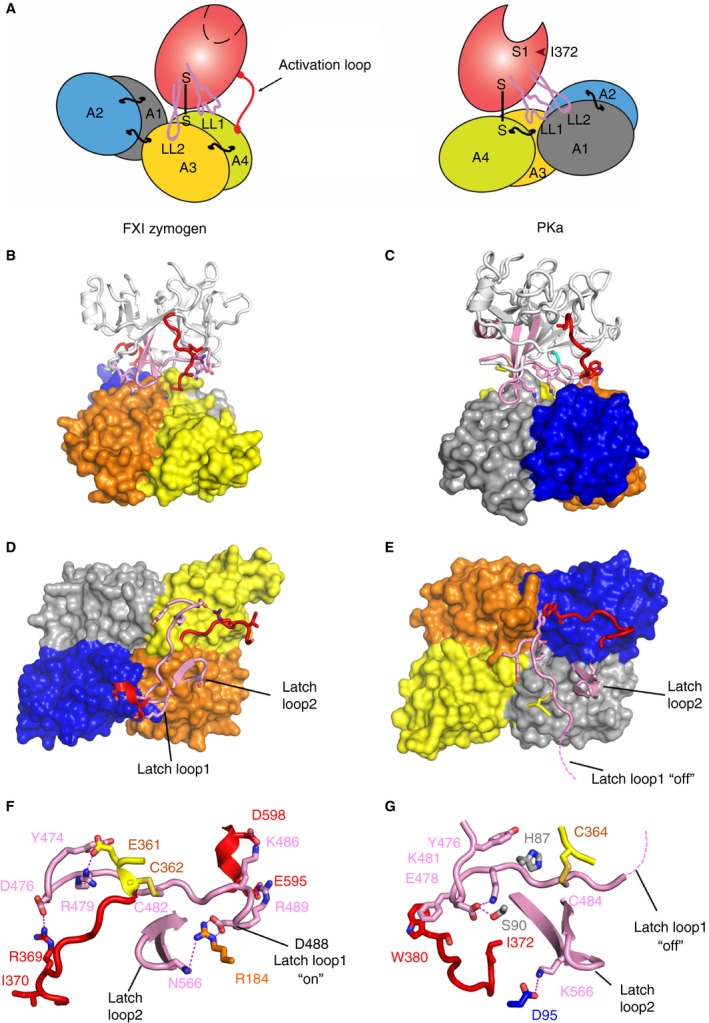
Comparison of full‐length PKa and FXI zymogen structures. (A) A schematic diagram of the domain organization observed in the FXI zymogen monomer (left) with two latch loops (LL1, LL2) located above the apple 3 domain compared to the PKa structure (right) with the latch loop 1 interacting with the apple 1 domain. (B) Cartoon diagram of the FXI zymogen crystal structure protease domain above a surface representation of the apple domains and (C) PKa with an equivalent orientation of the protease domain and color scheme to illustrate the large 180° relative rotation of the apple domains. The latch loops are pink and the activation loop red. (D) The FXI apple domains alone are shown as a surface representation with latch loop1 and loop2 (pink) and the activation loop (red) shown as cartoon. (E) The same figure of PKa shows the positions of the three loops at the interface between the protease domain illustrating 180°‐rotation compared to FXI with the latch removed and partially disordered (dotted line). Close‐up views of the latch loops “on” in FXI are shown in (F) with an equivalent latch loop orientation shown for PKa in (G). Key residues are highlighted as sticks including the Asp488 from latch loop 1, which covers the substrate binding pocket in the FXI zymogen. FXI, factor XI; PKa, plasma kallikrein.

A comparison of the full‐length structures of PKa with the FXI zymogen revealed a large unexpected conformational 180° rotational rearrangement of the intact apple domain disc relative to the protease domain (Fig. 4A–C). The PKa latch loop1 is a key pivot point for this relative rotation as it contains the Cys364‐Cys484 disulfide which anchors the protease domain to the apple domains after cleavage of the Arg371‐Ile373 peptide bond in the activation loop. A comparison between the FXI and PKa latch loop1 structure reveals the PKa *β*‐hairpin Glu478‐Lys481 ion pair is conserved as Asp476‐Arg479 in FXI. Latch loop1 has a 180° relative rotation from its position above the apple 3 and 4 domains in FXI where Asp476 interacts with Arg369 from the activation loop closer to the center of the disc inserting into a central pocket of PKa (Fig. 4B–E). Latch loop2 maintains a similar extended *β*‐hairpin structure in both PKa and FXI but whereas in FXI the interaction is with the apple 3 domain in PKa this equivalent *β*‐hairpin has moved by 40 Å to form interactions with the topside α‐helix of the PKa apple 1 domain (Fig. 4F,G).

Large relative rotational changes in the position of latch loop1 and 2 are consistent with previous biochemical data that FXI residues Asp488 and Asn566 act as a latch in the FXI zymogen structure covering the substrate factor IX (FIX) binding pocket in the apple 3 domain 24 (Fig. 4F). In the PKa structure, residues 488 to 493 containing the homologous region of the FXI latch are not observed in the PKa electron density and are assumed to be disordered and flexible, labeled as “latch off” in Fig. 4E,G. It is interesting to observe the direct interactions between the FXI activation loop residue Arg369 and the latch loop1 Asp476 (Fig. 4D,F).

### Hydrogen deuterium exchange protection differences between PK and PKa

**Figure 5:**
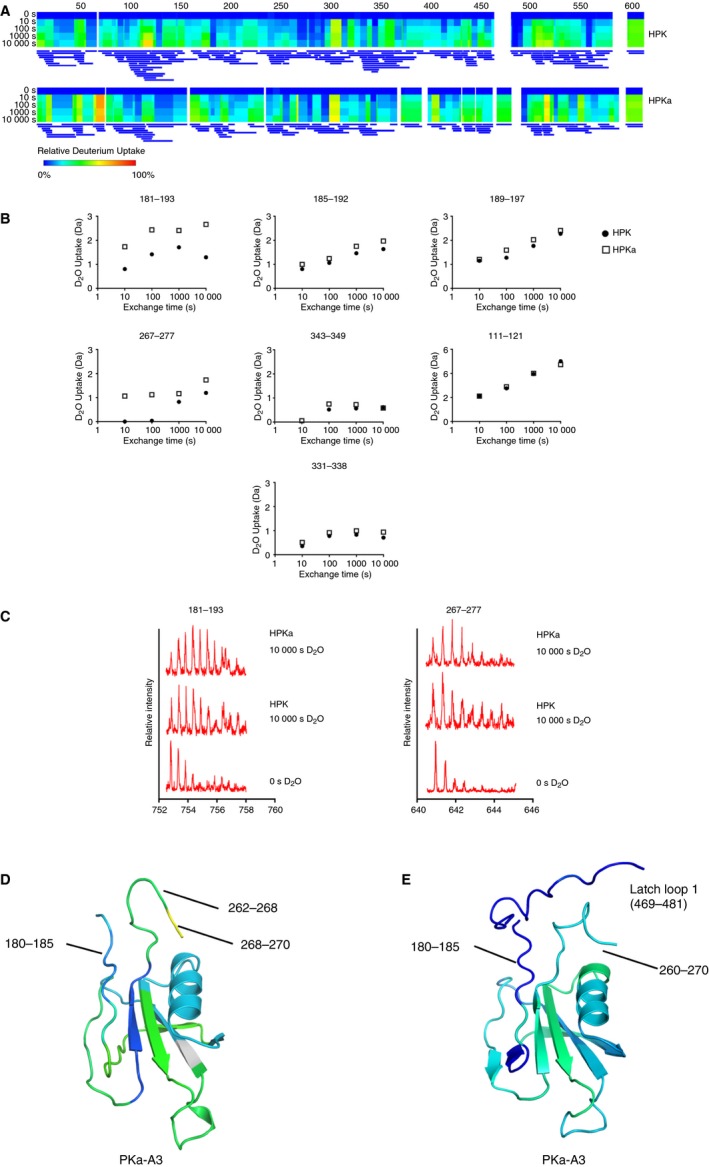
Hydrogen‐deuterium exchange (HDX) characterization of PK and PKa. (A) Heatmaps showing relative fractional uptake over exposure time via Dynamx Software. For PK, 150 peptide fragments were assigned, achieving 94.9% sequence coverage and a redundancy of 4.99. For PKa, 113 peptide fragments were assigned, achieving 94.7% sequence coverage and a redundancy of 3.14. (B) Deuterium uptake of specific peptides in PK and PKa. Deuterium uptake for peptides shared between the data sets was plotted over time of D_2_O exposure. Structural differences at the rotation site are consistent with HDX data presented, while structural consistencies remain largely the same and are supported in the HDX data. (C) Mass spectra of highlighted peptides that show differences in the PK and PKa apple 3 domain from 0 to 10 000 s D_2_O exposure. (D) Cartoon diagram of the PKa apple 3 domain crystal structure showing greater deuterium exchange for residues in the region of residues 180 to 185 (light blue) and 262 to 270 (green/yellow colored based on the heatmap) compared to (E) the PK zymogen apple 3 domain model with reduced deuterium exchange for the equivalent regions (dark blue). FXI, factor XI; PKa, plasma kallikrein.

To investigate whether large conformational differences exist between the PK zymogen and PKa in solution, HDX‐MS was performed on human plasma purified samples (termed HPK and HPKa). With this approach, the extent of deuterium exchange of backbone amide protons in HPK and HPKa was evaluated as a function of time. For HPK, 150 peptide fragments were assigned, achieving 94.9% sequence coverage and a redundancy of 4.99. For HPKa, 113 peptide fragments were assigned, achieving 94.7% sequence coverage and a redundancy of 3.14. Heat maps showing relative fractional deuterium uptake over exchange time were calculated with Dynamx Software. A residual heat map of each HPK and HPKa fragment from 0 to 10 000s is shown in rainbow scale (Fig. 5A), accompanied by the corresponding peptide coverage map below. Residues with no peptide coverage are left blank. Figure 5B shows the examples of deuterium uptake for some identical peptides in HPK and HPKa, with significant differences in deuterium uptake observed for residues 181 to 193 and 267 to 277, implying that these residues on the upper surface of the apple 3 domain are more exposed in HPKa compared to HPK. In addition, example peptides are shown that have little difference between HPK and HPKa such as peptide 111 to 121. These regions appear structurally congruent between the PK zymogen model and PKa structure. The complete set of deuterium uptake plots for all the assigned peptides in HPK and HPKa is included as Figs. S1 and S2.

Solvent accessibility differences between the PK zymogen model and PKa structure were calculated using the program STRIDE 38 and overall, the HDX‐MS results are consistent with the accessibility data. Notably, a significant change occurs for residues 267 to 277 in the loop connecting the apple 3 and apple 4 domain, which is folded toward the center of the apple domain disc and buried by the protease domain in the PK zymogen compared to PKa where a radically different conformation of this loop exposes residues 262 to 268 onto the surface of the disk adjacent to residues 180 to 185, which also show an increase in accessibility (Fig. 5C). A 3D projection of HDX data at the 10 000 s exchange time mapped to the structure of PKa and the PK zymogen model apple 3 domain is shown in Fig. 5D,E. Thus the HDX data support a model whereby the apple 3 domain top surface residues become exposed when PK is activated to PKa. The heat map reveals differences in the region around residue 75 in the apple 1 domain that are more surface exposed in HPKa compared to HPK. This region is not predicted to have changes in surface exposure from the structural comparison of PKa with the PK model and may reflect the reduced peptide coverage in this region compared to the apple 3 domain or an incomplete description of the PK model.

## Discussion

We present the first example of a full‐length crystal structure for PKa at 1.3 Å resolution, which defines the detailed organization of the active protease domain and its relationship to the four ancillary apple domains. A comparison with a 2.6‐Å FXI zymogen structure revealed significant conformational differences in the apple 4 domain defining the molecular basis of the monomeric PKa structure versus the FXI dimer. A major conformational difference is also observed in the apple 3 domain with residues from the loop connecting the apple 3 to the apple 4 domain becoming exposed in PKa as opposed to being buried in the center of the apple domain disc in the zymogen form of FXI. It is tempting to speculate that PK may undergo conformational rearrangements upon activation based on the comparison of the FXI and PKa structures. This hypothesis is supported by HDX mass spectrometry measurements on plasma purified human PK and PKa determining that regions of the apple 3 domain have increased surface exposure in PKa compared to the zymogen PK. The biological significance of the increased exposure of residues on the upper surface of the apple 3 domain is unknown for PK but has been proposed to occur in FXI to regulate substrate binding 21, 24. Here it was shown that FXIa has an exosite for the FIX Gla domain involving residues 183 to 185 from the FXIa apple 3 domain, which is cryptic in the FXI zymogen 25, 36, 37. Consistent with this the FXI zymogen structure shows Arg184 is buried and forms a salt bridge with Asp488 from the catalytic domain (LL1), rendering it unavailable for binding to the FIX Gla domain 19, 21. By comparison with the apple 3 domain the apple 1 domain does not experience a large change in conformation.

A comparison of PKa with the FXI structure revealed a large conformational 180° rotational rearrangement of the intact apple domain disc relative to the protease domain. Low‐resolution structural studies and biochemical data comparing the homologous FXI and active form FXIa have suggested that a large conformational shape change occurs upon activation 39, 40. Future studies will be required to determine a crystal structure of the PK zymogen to establish whether this mirrors the conformation observed for the FXI zymogen and to determine whether the PKa apple 3 domain is utilized for substrate recruitment as it is in FXIa. Other substrates reported for PKa include the integral membrane proteins protease activated receptor (PAR), PAR‐1, and PAR‐2 implicated in regulation of blood‐brain barrier integrity 41 and platelet activation 42. It is interesting to note that the P1‐P1′ cleavage site Arg‐Ser in the PAR‐1 and PAR‐2 sequences also occurs in the classical PKa substrate HK to release bradykinin 43. The cleavage of extracellular matrix proteins by PKa has also been described in the astrocyte secretome although the precise location of the cleavage sites and involvement of the apple domains in substrate selection remains unknown 44. The interaction of PK and FXI with cell receptors has been characterized and the apple domains have been reported to be implicated although the precise binding sites remain unknown 21, 45.

Overall these new structures provide the first insight into the active conformation of the apple domain containing protease PKa and generate a scaffold for development of future medicines targeting PKa in diverse inflammatory, thrombotic, and vascular disorders 12, 13.

## Author contributions

CL purified, crystallized, and determined the structure of PKa. MP and ID generated PK expression constructs and contributed to the manuscript. GH crystallized and determined the structure of FXI. KMV and RL performed and analyzed HDX experiments. JE initiated and designed research, analyzed results, and wrote the paper with ID, KM, and RL.

## Disclosures of Conflict of Interest

The authors declare no conflicts of interest.

## Supporting information


**Fig. S1.** HPK raw time‐dependent deuterium uptake chart for each peptide fragment.

**Fig. S2.**  HPKa raw time‐dependent deuterium uptake chart for each peptide fragment.
