# Supplementary figures and images for: Plasma kallikrein structure reveals apple domain disc rotated conformation compared to factor XI
Source: J Thromb Haemost. 2019 Mar 19;17(5):759–70. doi: 10.1111/jth.14418 (PMC6899681; doi:10.1111/jth.14418)

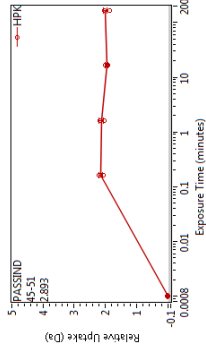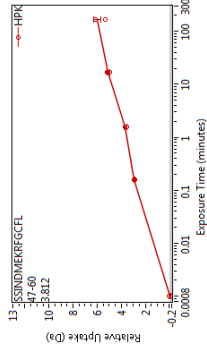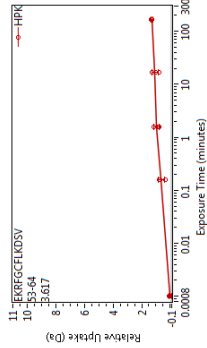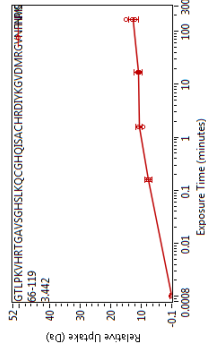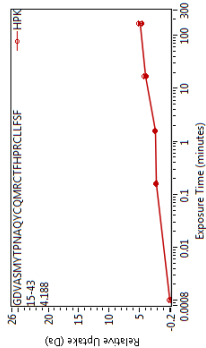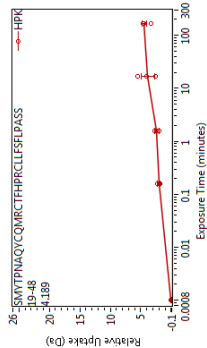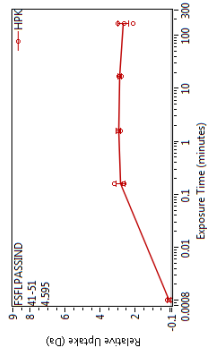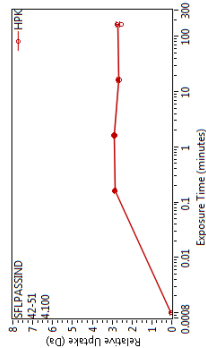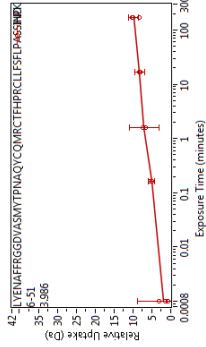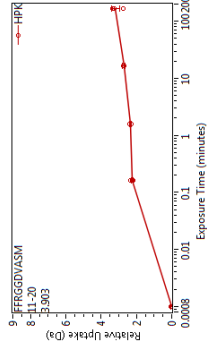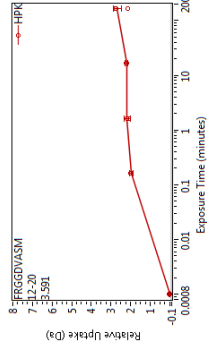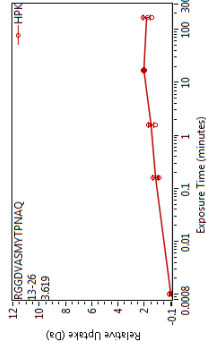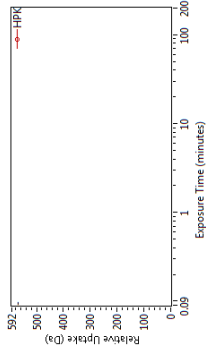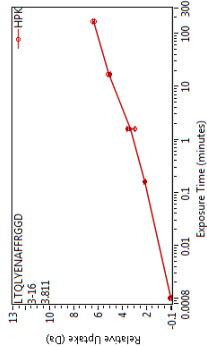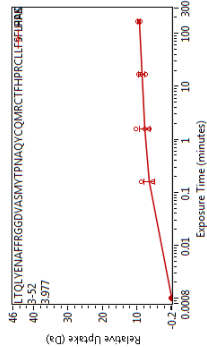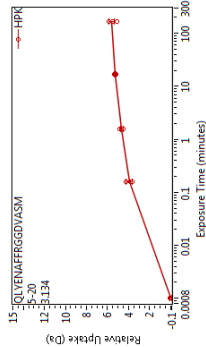

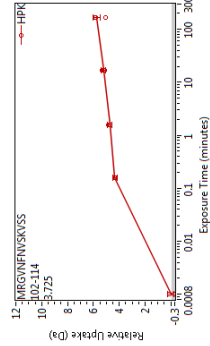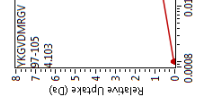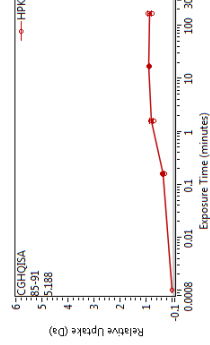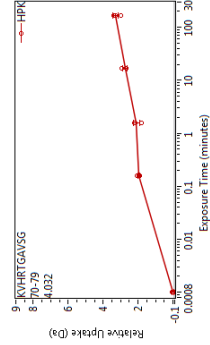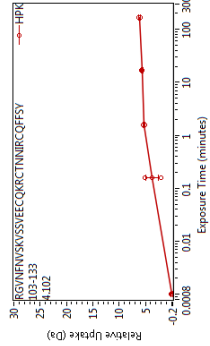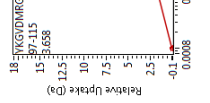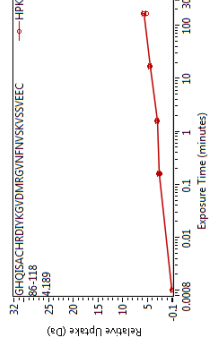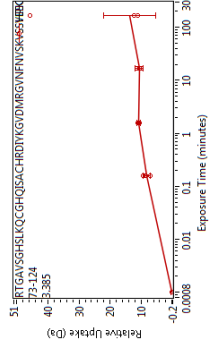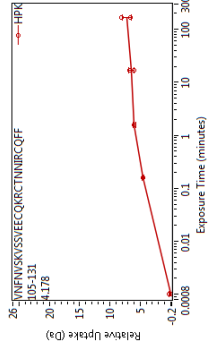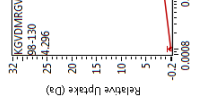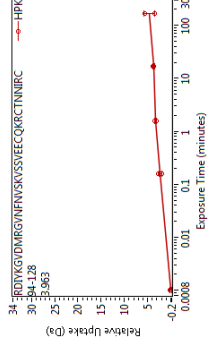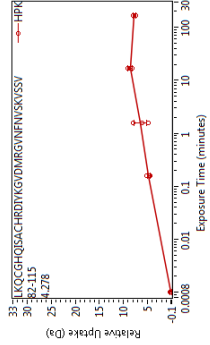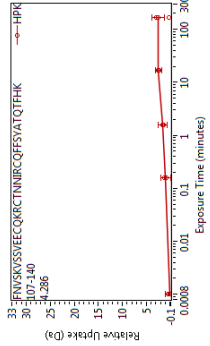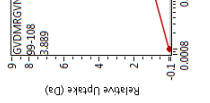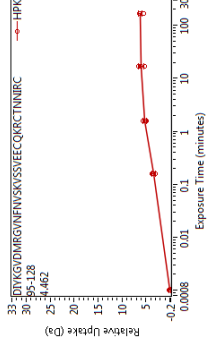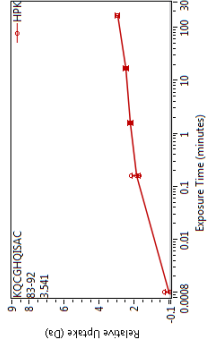

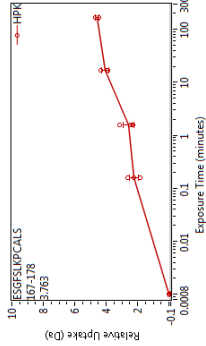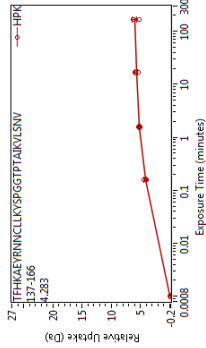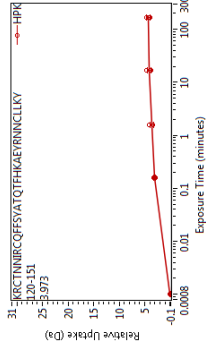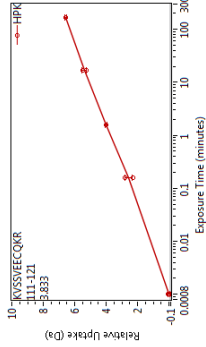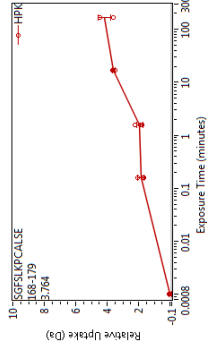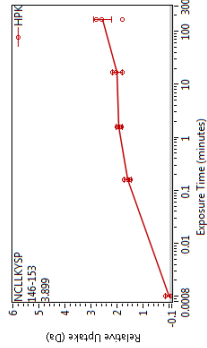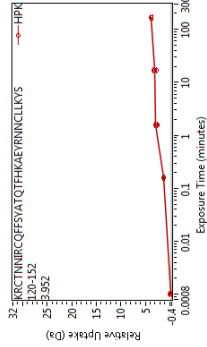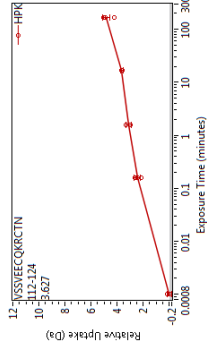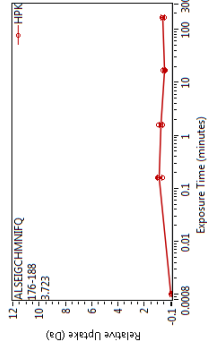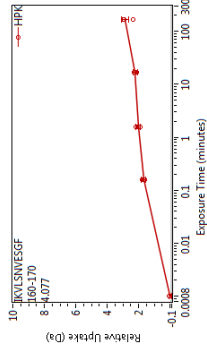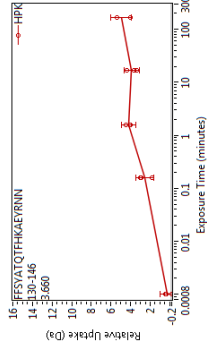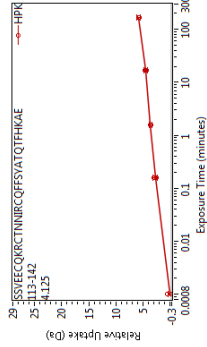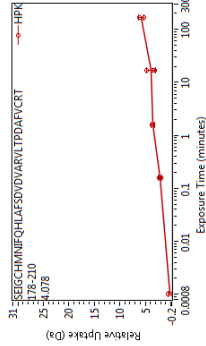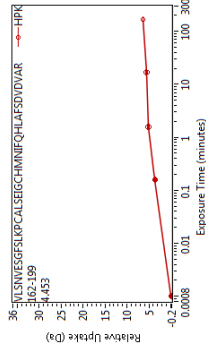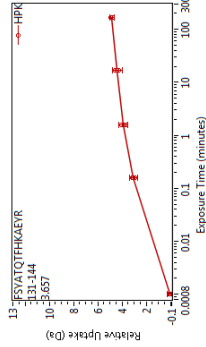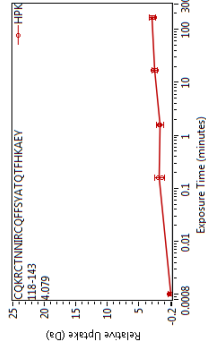

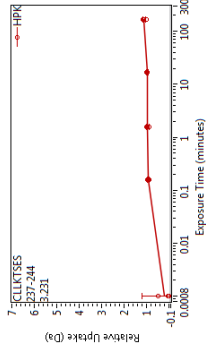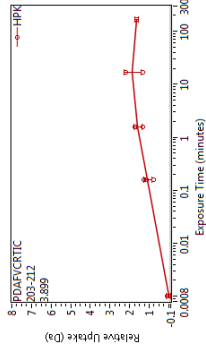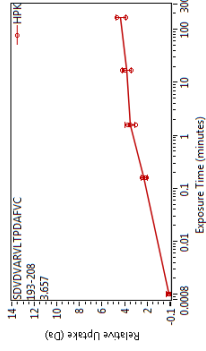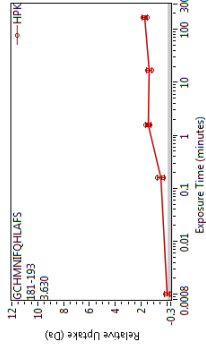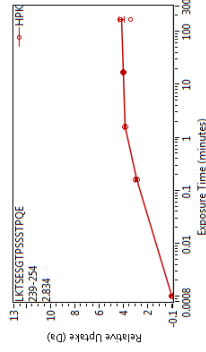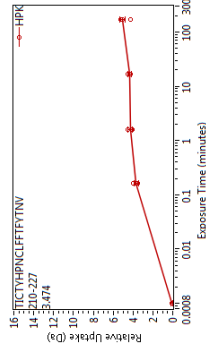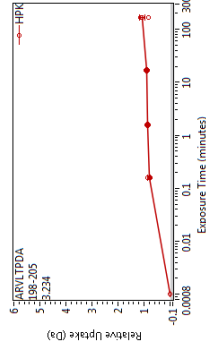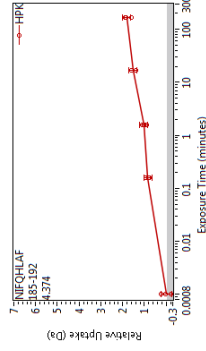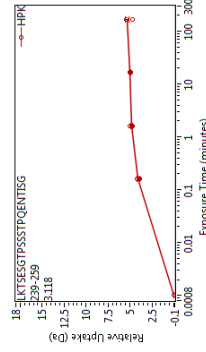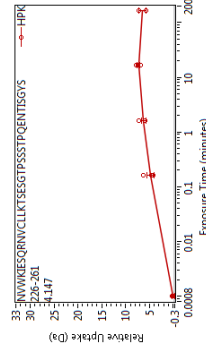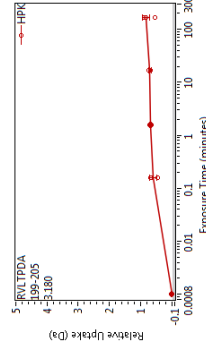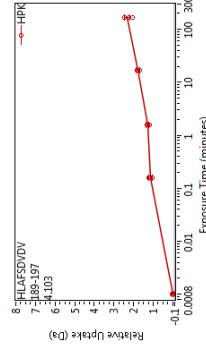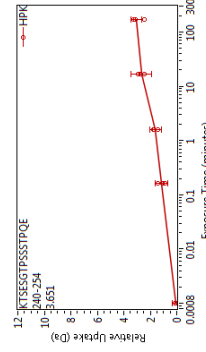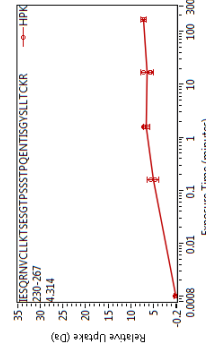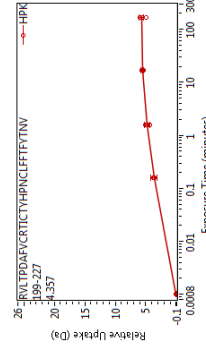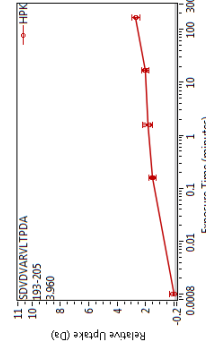

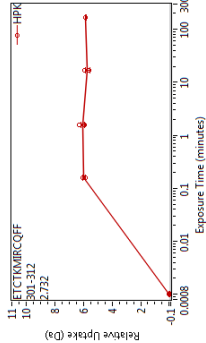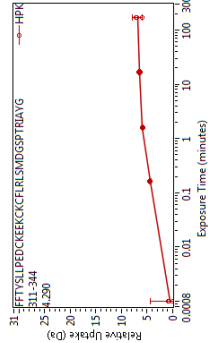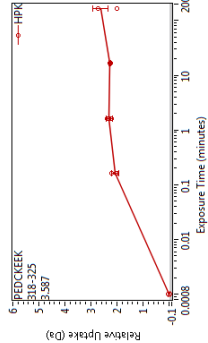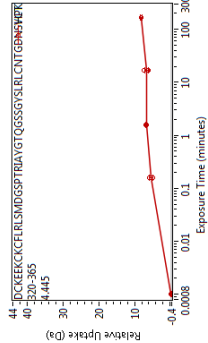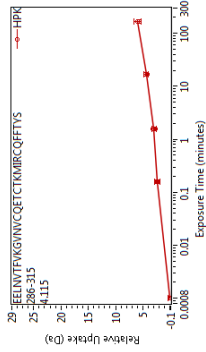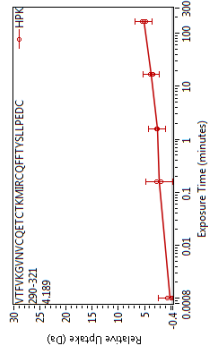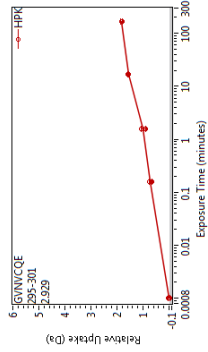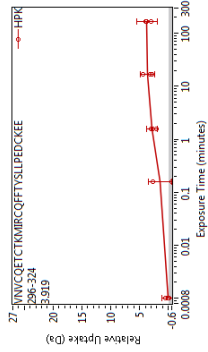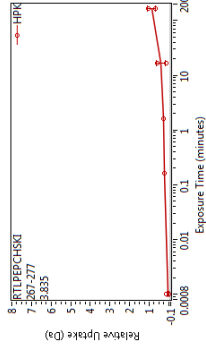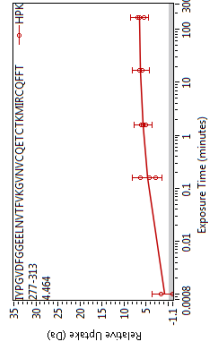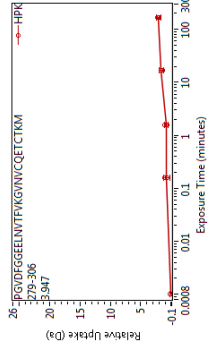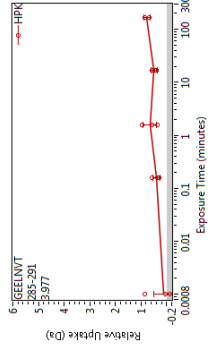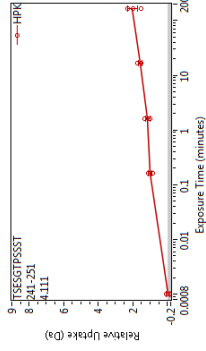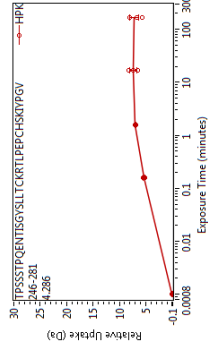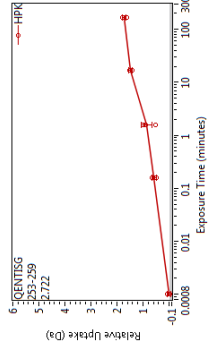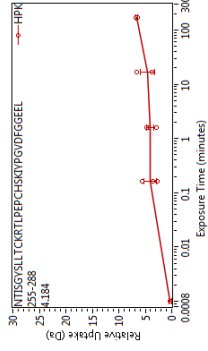

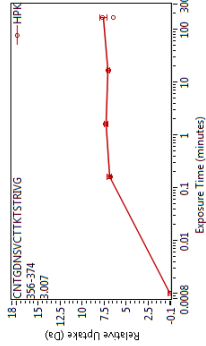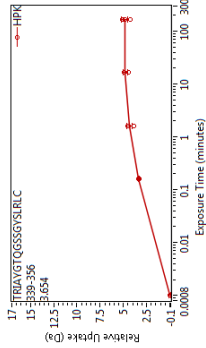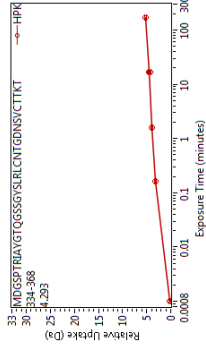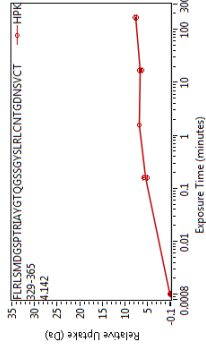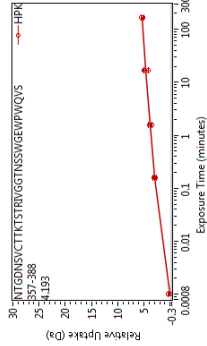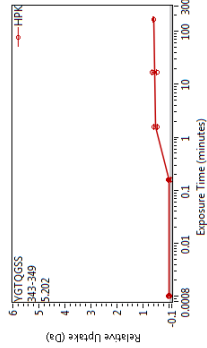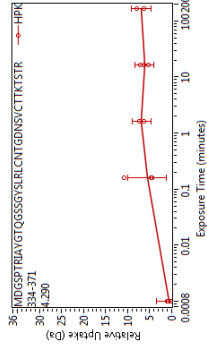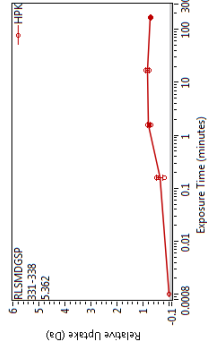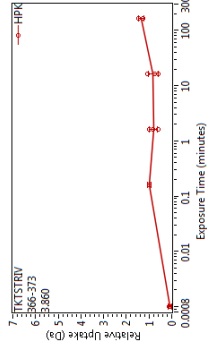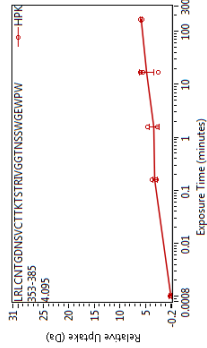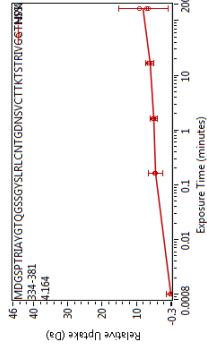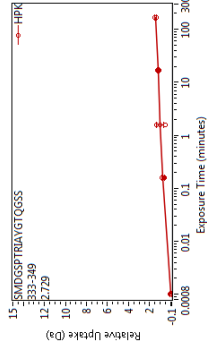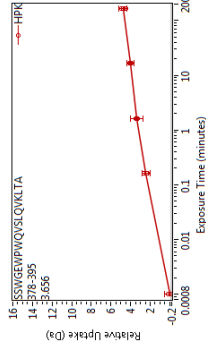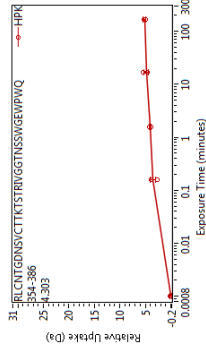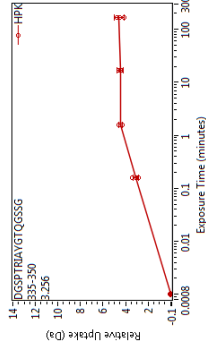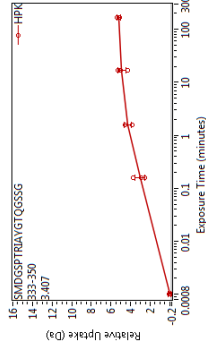

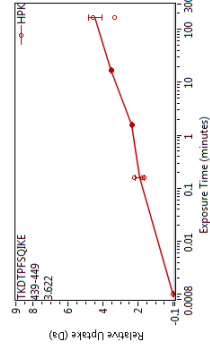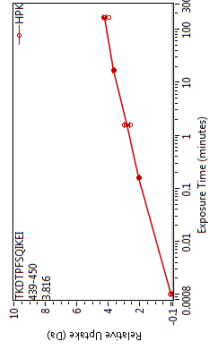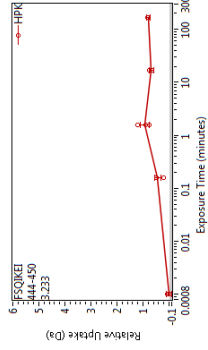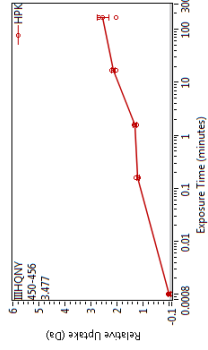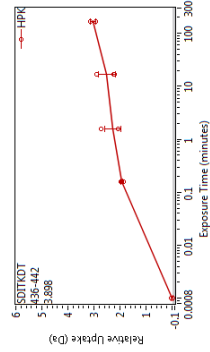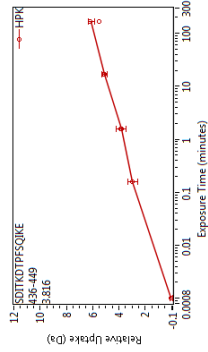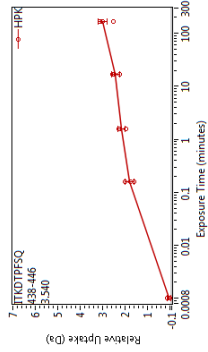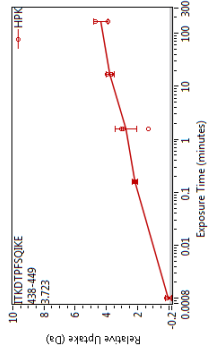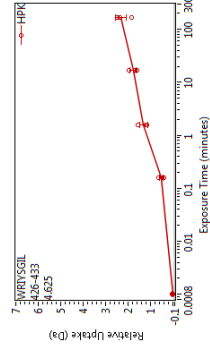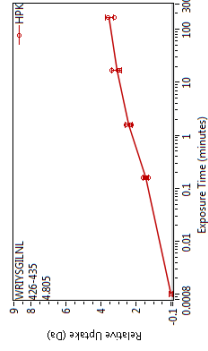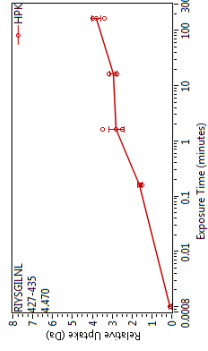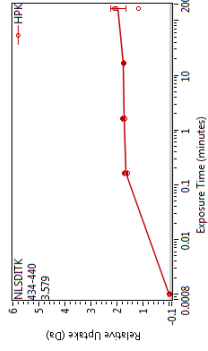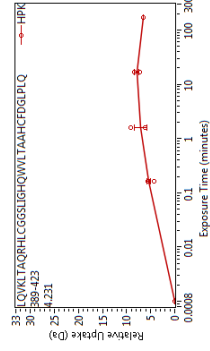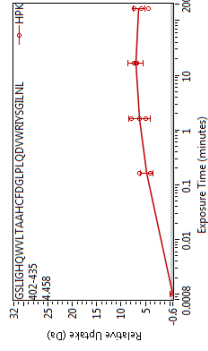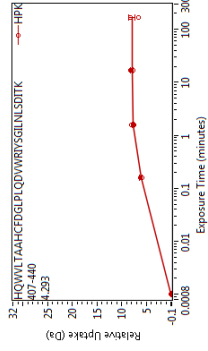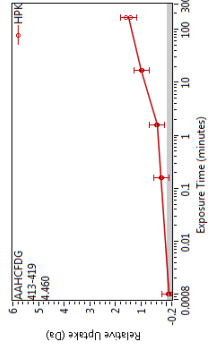

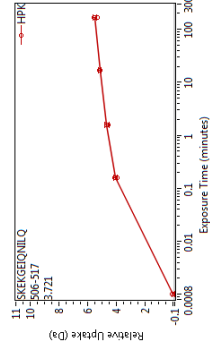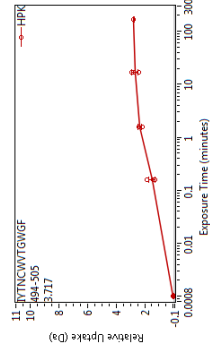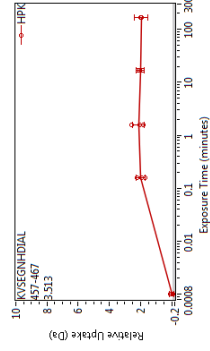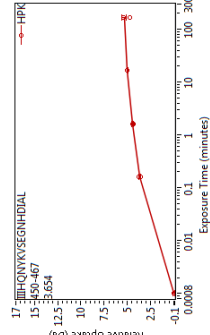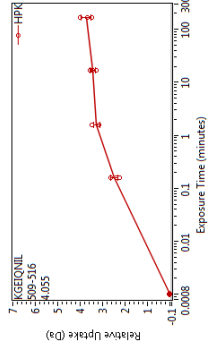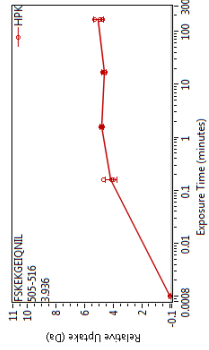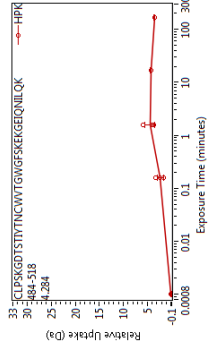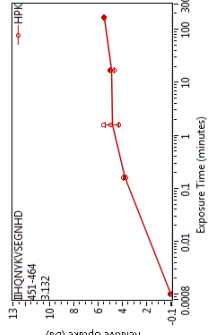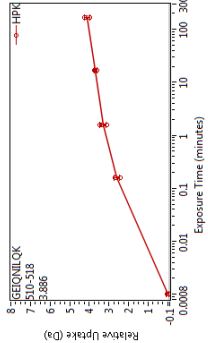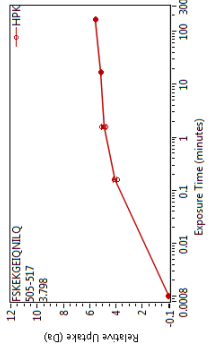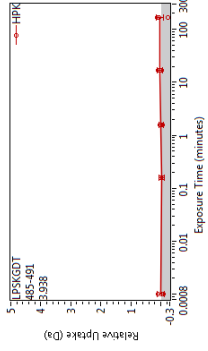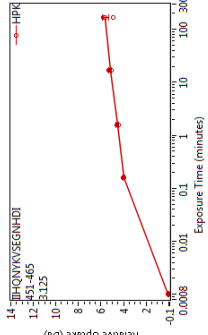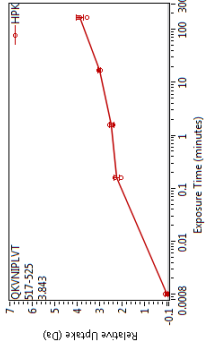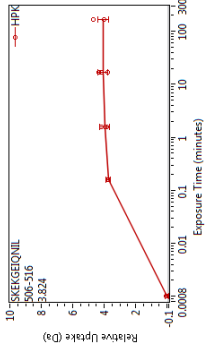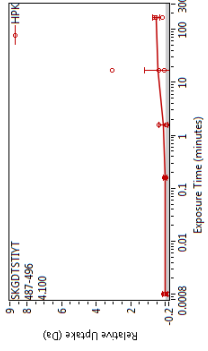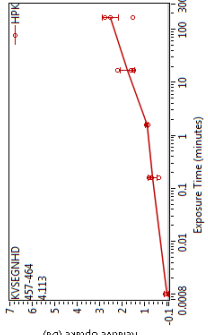

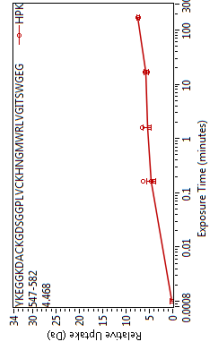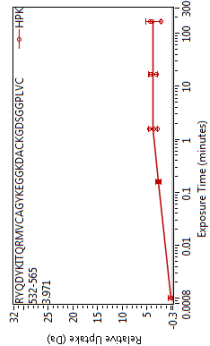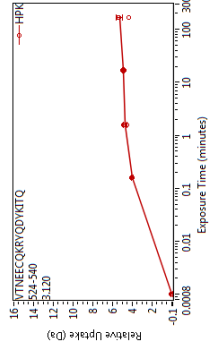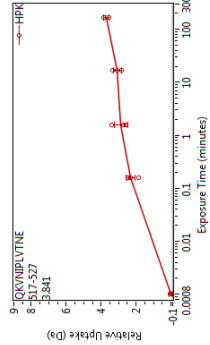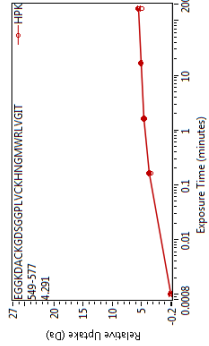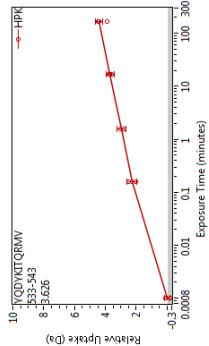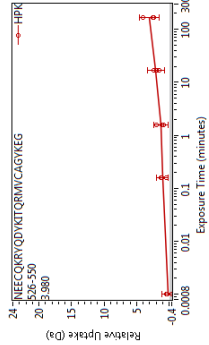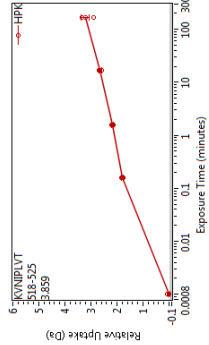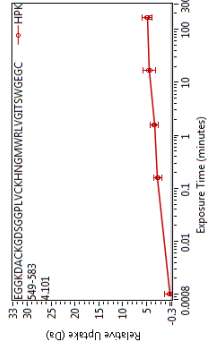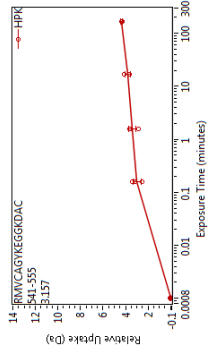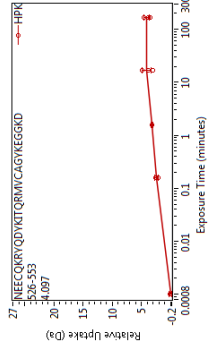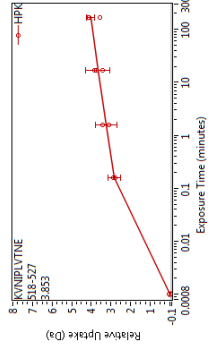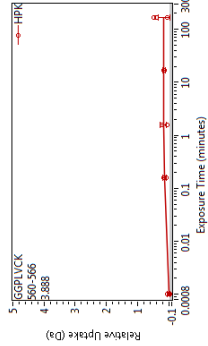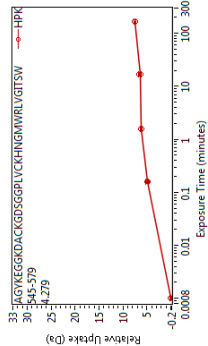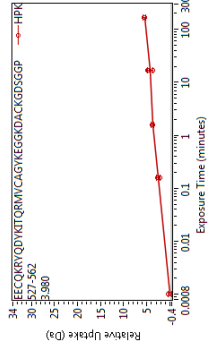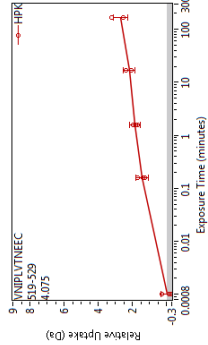

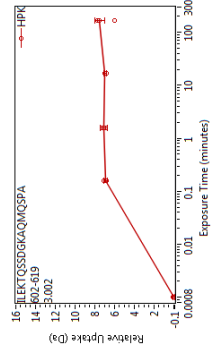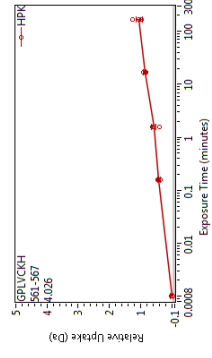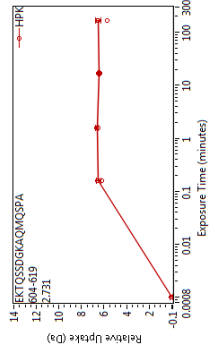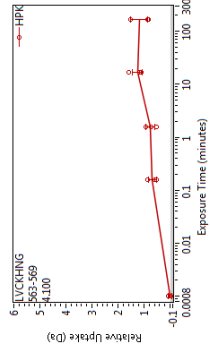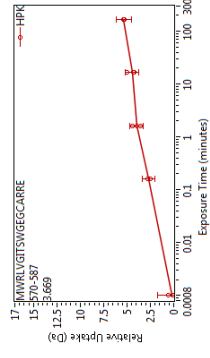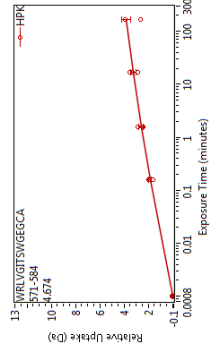

Supplement: Supplementary file 1 — Fig. S1. HPK raw time‐dependent deuterium uptake chart for each peptide fragment. [file JTH-17-759-s001.pdf]

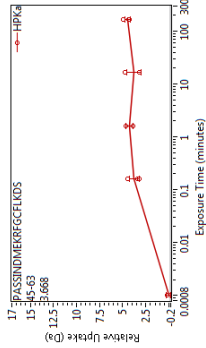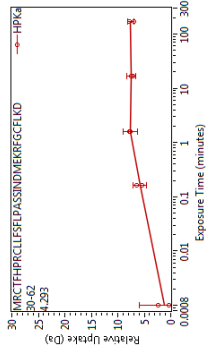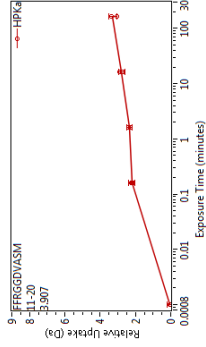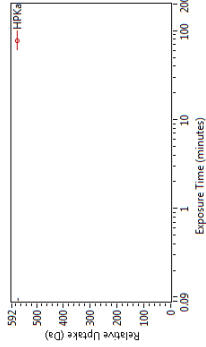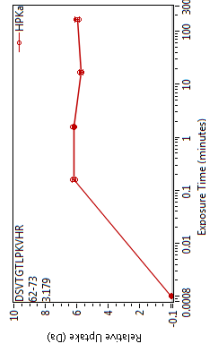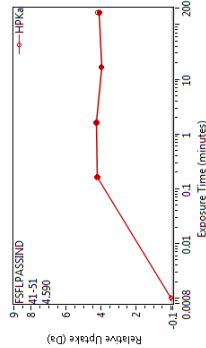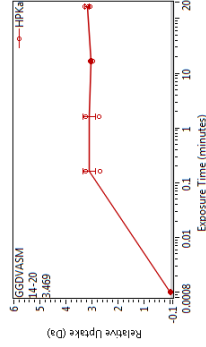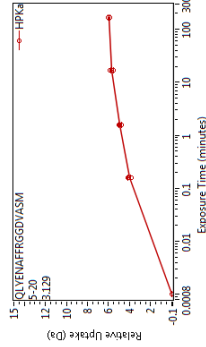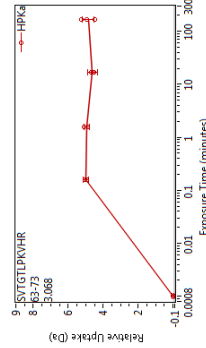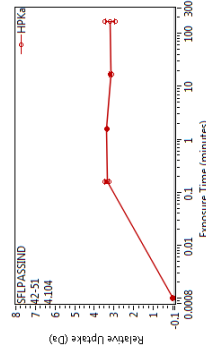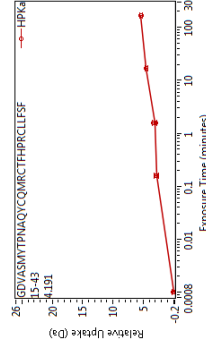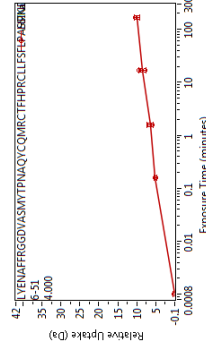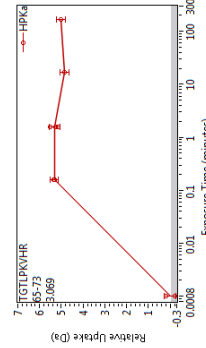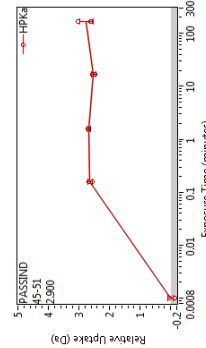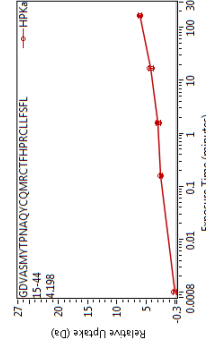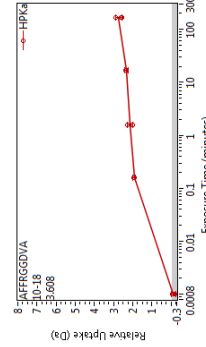

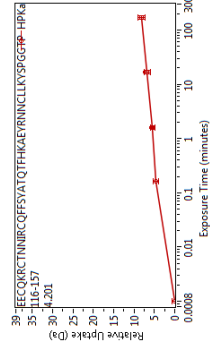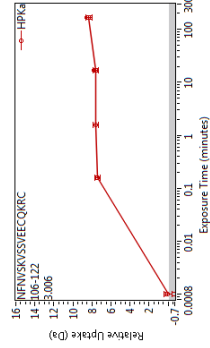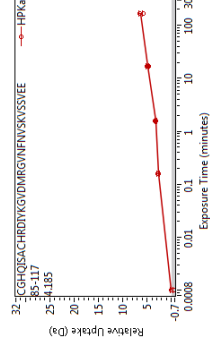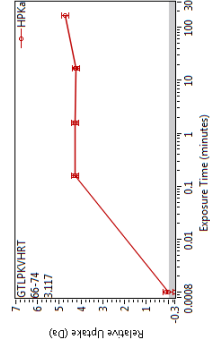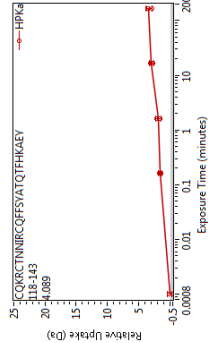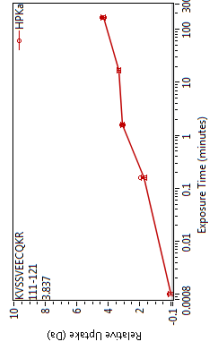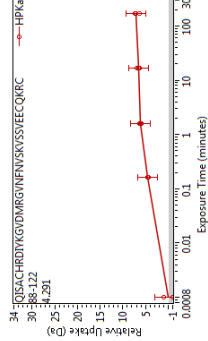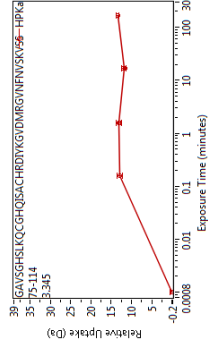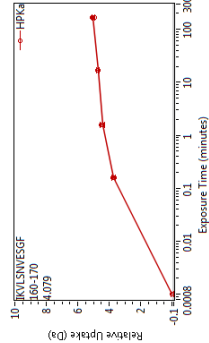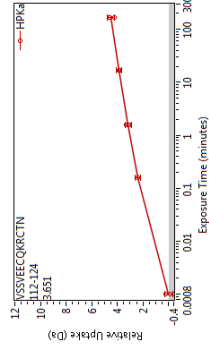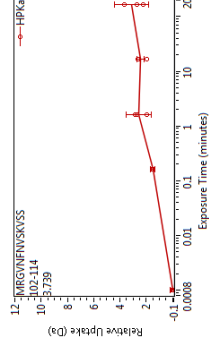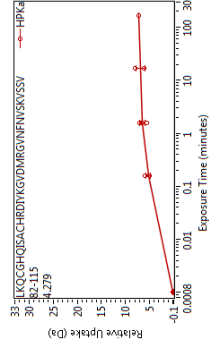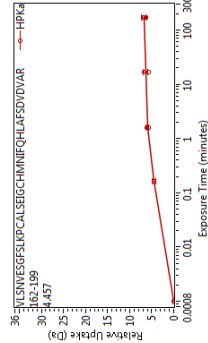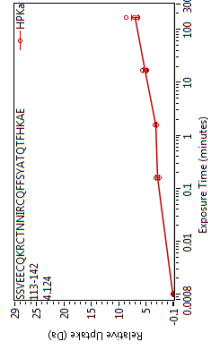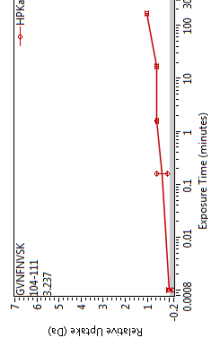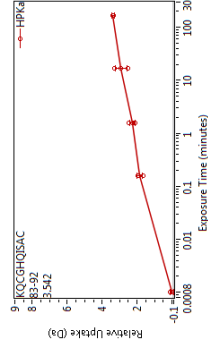

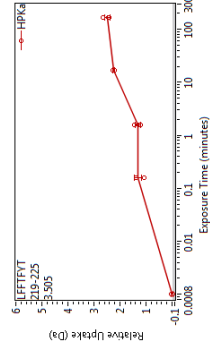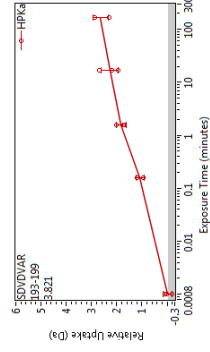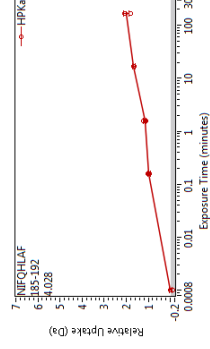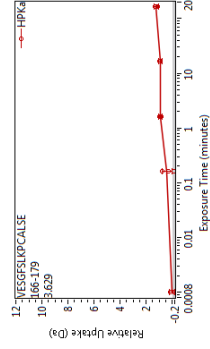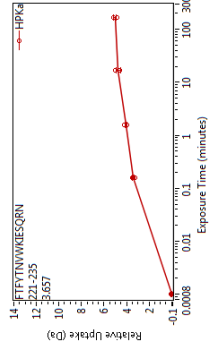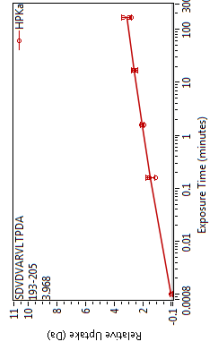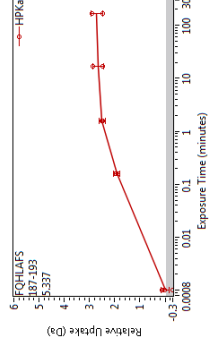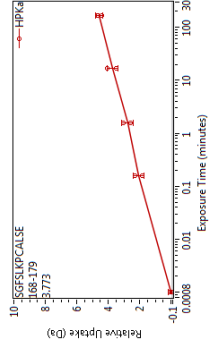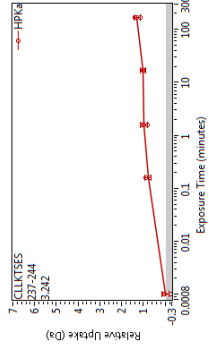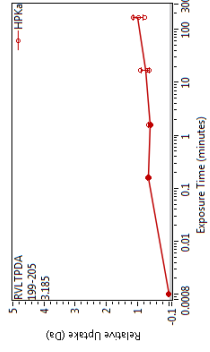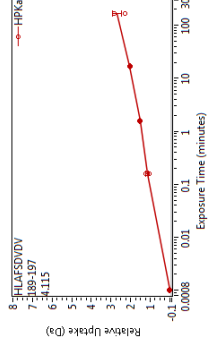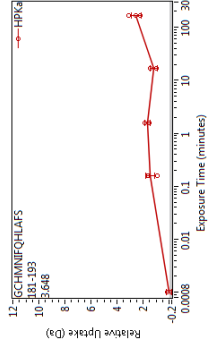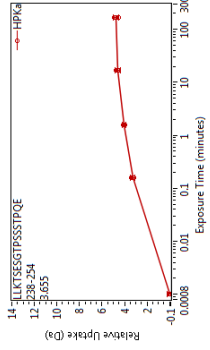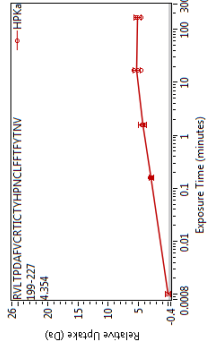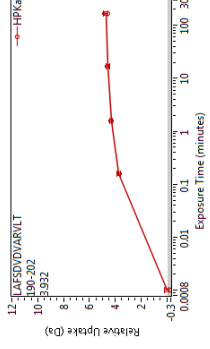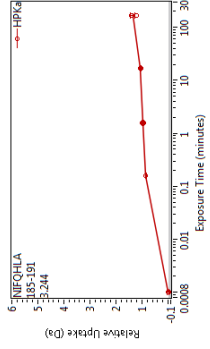

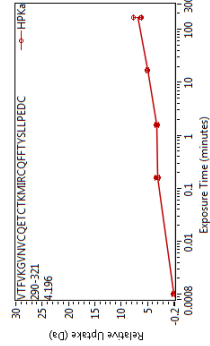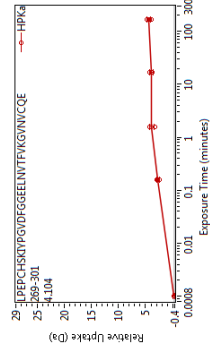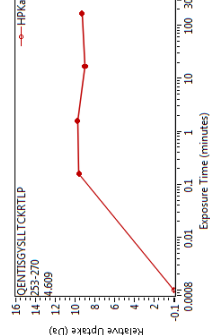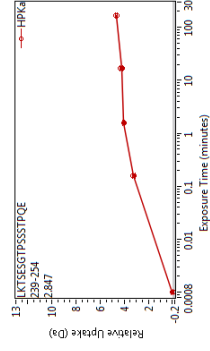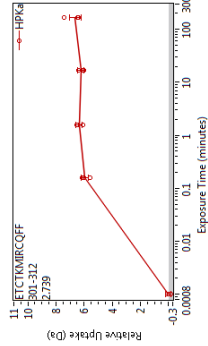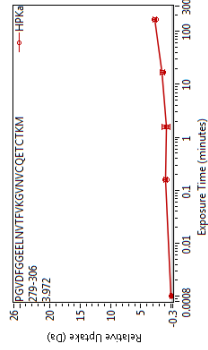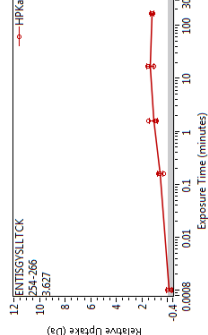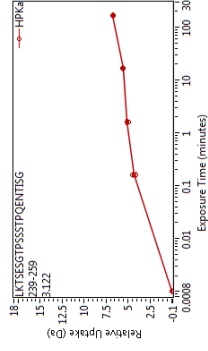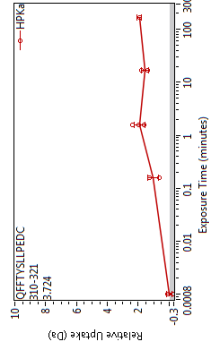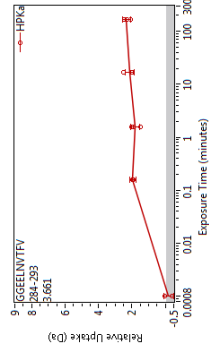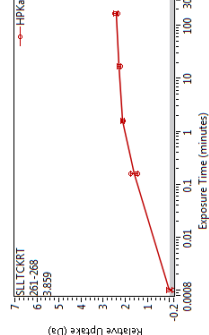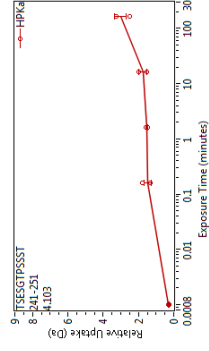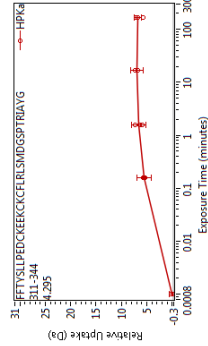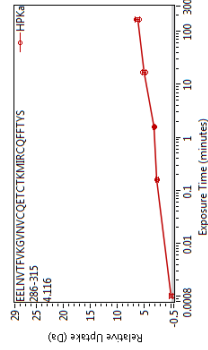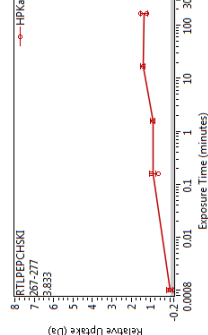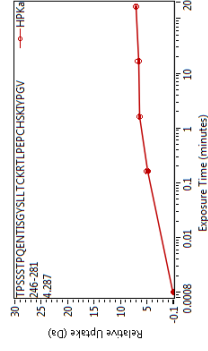

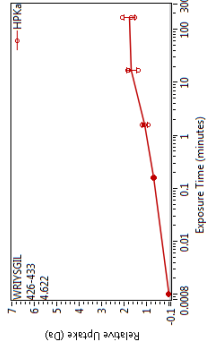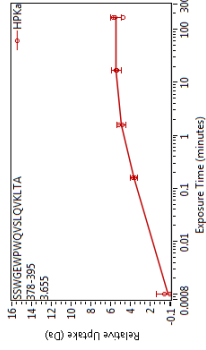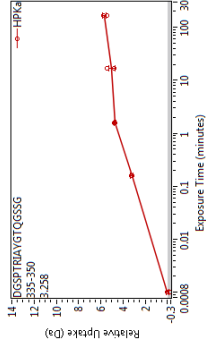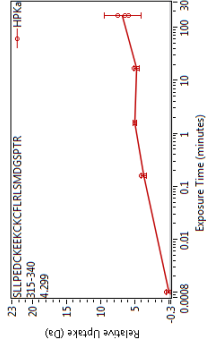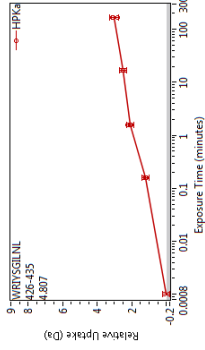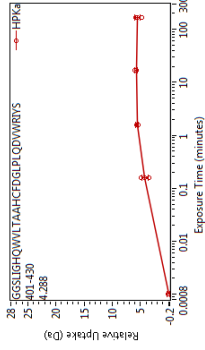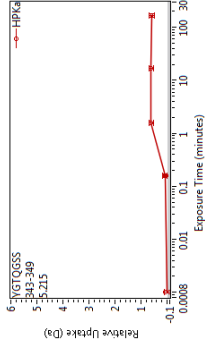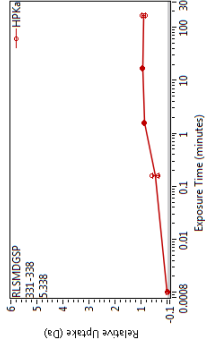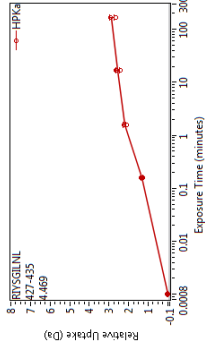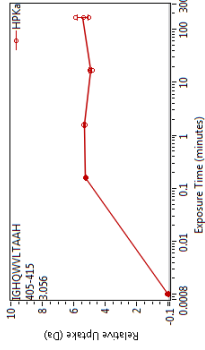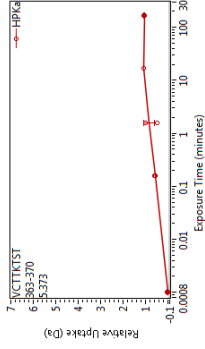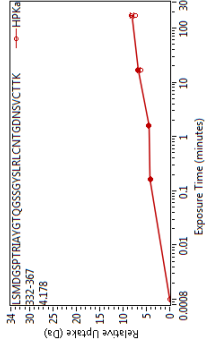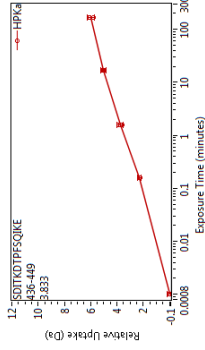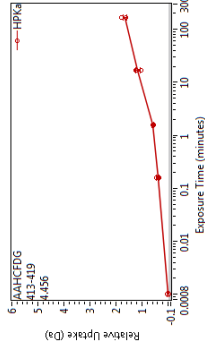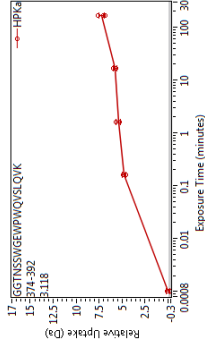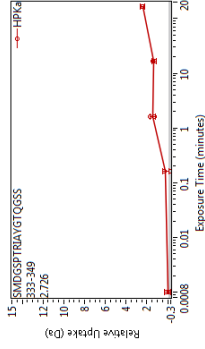

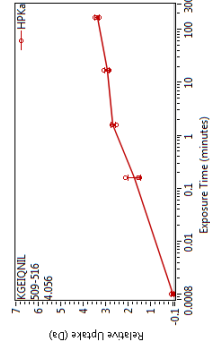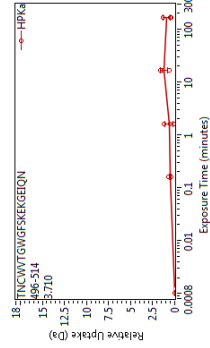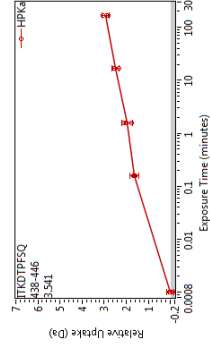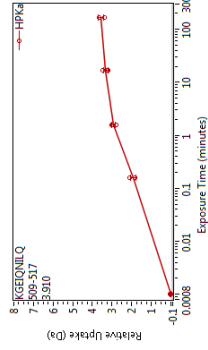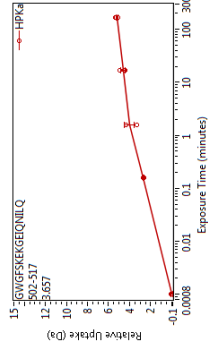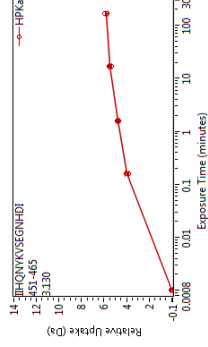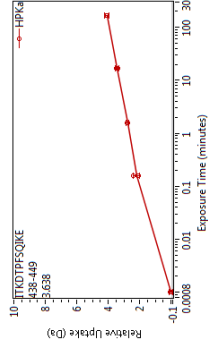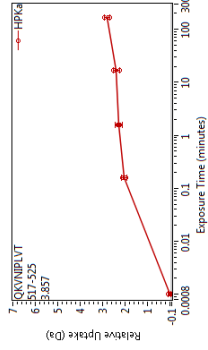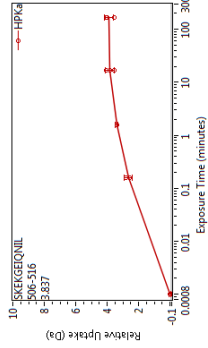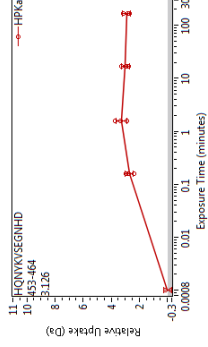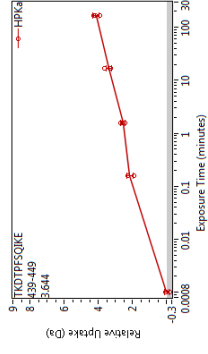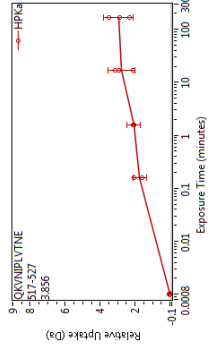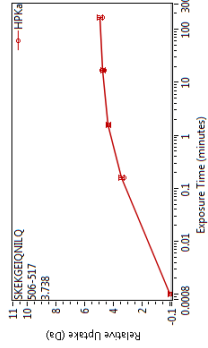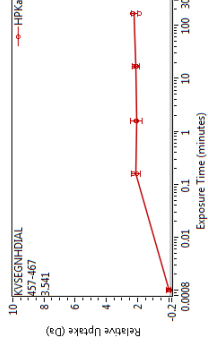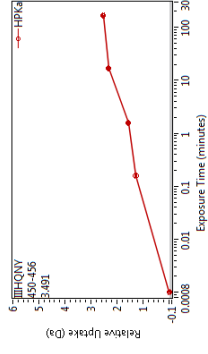

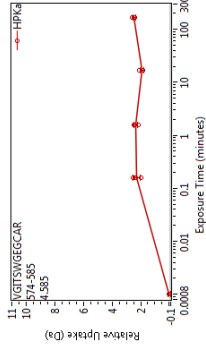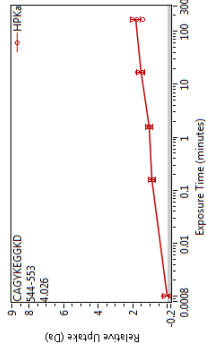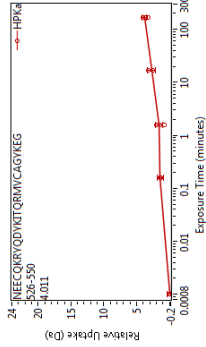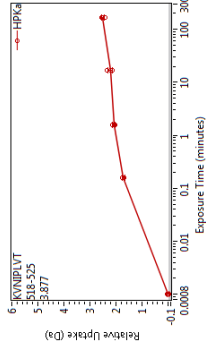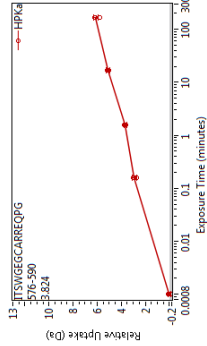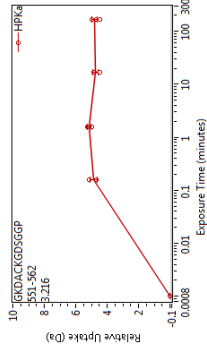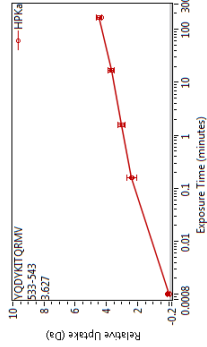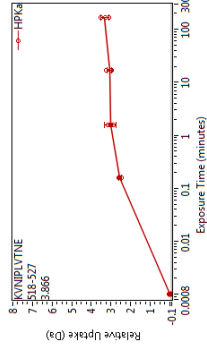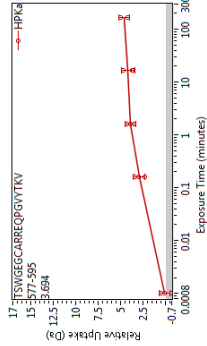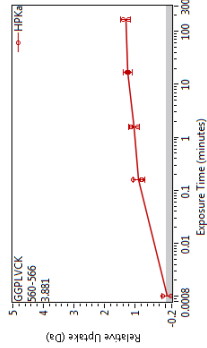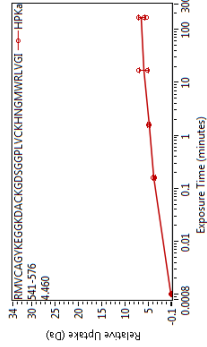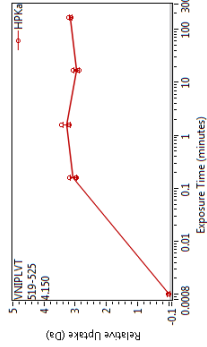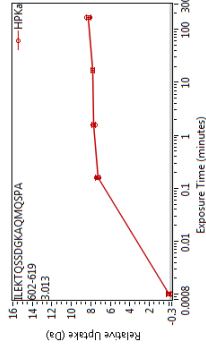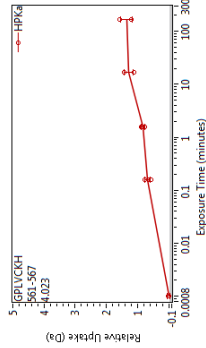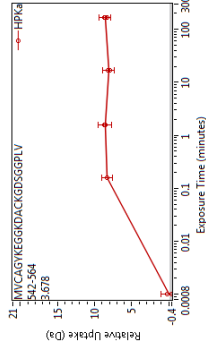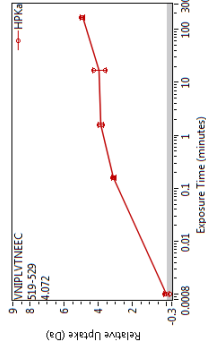

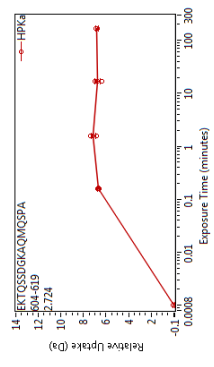

Supplement: Supplementary file 2 — Fig. S2. HPKa raw time‐dependent deuterium uptake chart for each peptide fragment. [file JTH-17-759-s002.pdf]
